# Clinical significance of self-descriptive apathy assessment in patients with neurological form of Wilson’s disease

**DOI:** 10.1007/s10072-021-05366-0

**Published:** 2021-06-14

**Authors:** Marcin Leśniak, Magdalena Roessler-Górecka, Anna Członkowska, Joanna Seniów

**Affiliations:** 1 Faculty of Psychology, University of Warsaw, Warsaw, Poland; 2 Institute of Psychiatry and Neurology, Sobieskiego 9, 02-957 Warsaw, Poland

**Keywords:** Wilson’s disease, Apathy syndrome, Psychopathology, Cognitive impairment, Self-description

## Abstract

**Background and aim:**

Apathy is one of the neuropsychiatric symptoms of Wilson’s disease (WD) which typically affects the brain’s fronto-basal circuits. Lack of agreed diagnostic criteria and common use of self-description assessment tools lead to underestimation of this clinical phenomenon. The aim of this study was to investigate whether subjective and informant-based clinical features of apathy in patients with WD enable clinicians to make a valid diagnosis.

**Methods:**

Multiple aspects of goal-oriented behavior were assessed in 30 patients with the neurological form of WD and 30 age-matched healthy participants using two questionnaires, the Lille Apathy Rating Scale (LARS) and the Dysexecutive Questionnaire (DEX). Both included a self-descriptive and a caregiver/proxy version. Cognitive functioning was estimated with the use of Addenbrooke’s Cognitive Examination-Revised.

**Results:**

Patients obtained significantly worse scores on all clinical scales when more objective measures were considered. Features of apathy and executive dysfunction were revealed in patients’ caregiver versions of LARS and DEX, which may indicate poor self-awareness of patients with WD. Roughly 30% of participants were likely to present with clinically meaningful symptoms, independent of cognitive dysfunction.

**Conclusions:**

Methods relying on self-description appear inferior to informant-based scales when diagnosing apathy. More objective criteria and measurement tools are needed to better understand this clinical syndrome.

## Introduction

Wilson’s disease (WD) is a rare, autosomal recessive, inherited disease in which mutation of the ATP7B gene on chromosome 13 induces an adenosine triphosphate (ATP) synthesis deficiency [1, 2]. As a consequence, excess copper accumulates in various organs, including the liver, cornea, kidney, brain, and, especially, the basal ganglia. Clinical presentation may involve predominantly hepatic, neurological, or psychiatric symptoms. Neuropsychological profiles of the neurological form of WD are not fully investigated since this population is very heterogenous [3, 4]. Patients with brain involvement, in addition to movement disorders and other neurological symptoms, frequently show impairment of basic cognitive functions (memory, attention, and visuospatial processing) and executive functions of varying intensity [5, 6]. These symptoms may be accompanied by mood disorders, personality change, impulsivity, irritability, and other behavioral abnormalities, as well as psychosis [7]. To date and to the best of our knowledge, no research focused on apathy syndrome in WD has been published, although it is commonly reported in pathology-comprising basal ganglia and their neuronal loops with prefrontal cortex [8, 9].

Apathy is frequently revealed in neurodegenerative diseases with basal ganglia dysfunction, such as Parkinson’s disease (PD) [10], progressive supranuclear palsy [11], Huntington’s disease [12], and focal lesions due to vascular or traumatic brain injury, especially if the caudate, internal pallidum, and medial-dorsal thalamic nuclei are damaged [8, 13]. Apathy is associated with significant problems, including reduced independence in everyday life, poor response to treatment, and caregiver distress [14]. Clinically, however, apathy continues to be under-recognized, partly due to the lack of universal diagnostic criteria and tools [15].

Apathy has been defined either as an individual symptom or as a complex syndrome with diminished drive and motivation and/or action initiation as the main feature [16]. As a consequence, prominent reduction of goal-oriented behavior is clinically observed. According to Marin’s definition [17], this motivational deficit is not attributable to diminished level of consciousness, cognitive impairment, or emotional distress. Although some symptoms overlap, the apathetic condition can also manifest independently of depressive disorder [17].

According to some authors, apathy syndrome may be fractionated into components or affected domains: emotional, cognitive, and behavioral [16], incorporating conditions such as poor emotional responsiveness, indifference, lack of concern and intellectual curiosity, and reduced voluntary goal-directed behavior [18]. Multidimensional attitude towards the differential diagnosis of apathy has been frequently postulated. Previous authors have indicated the need for diagnostic tools designed for specific clinical symptomatology, allowing description of apathy profiles or even subtypes, independent of physical disability or anosognosia (i.e., by obtaining additional data from informants) [18, 19].

Apathy in WD may be a serious clinical problem, worthy of attention during assessment and treatment as well as during psychological work with patients’ families. However, due to frequent metacognitive dysfunction and limited self-awareness of this patient population, relying on self-report methods may pose a considerable threat to the validity of such an assessment.

Consequently, the main goal of this study was to investigate whether subjective and informant-based clinical features of apathy in patients with the neurological form of WD are consistent enough to allow clinicians draw practical conclusions.

We have taken the hypothesis of occurrence of apathy in WD patients not only on the basis of our clinical experience, but also relying on literature indicating that it is common when the executive (initiating and control) system for goal-directed behavior is altered [20]. The executive disorders are typical when neuronal prefrontal-basal ganglia loops are dysfunctional, as a feature of WD. Hence, apathy was assessed in the context of executive functioning, especially in regards to its metacognitive and behavioral aspects.

We also aimed to evaluate relationships between apathy and cognitive functioning to determine whether these entities are separable or co-occurring, which could suggest mutual pathomechanisms.

## Methods

### Participants

Thirty patients with chronic, neurological WD admitted to the Department of Neurology were enrolled in the study (Table 1). Inclusion criteria were (1) diagnosis of WD based on clinical symptom assessment, laboratory tests, and genetic analysis [21], (2) age of 18–65 years, and (3) duration of illness no shorter than 4 years (with stable medical condition and adjusted pharmacotherapy). Exclusion criteria were (1) severe somatic state (significantly hindering psychological examination), (2) limited consciousness, (3) overt affective syndromes (i.e., depression, mania), (4) coexisting neurological disorders other than WD, (5) poor compliance (i.e., irregular medication), and (6) severe communication or perception disorders that may interfere with performance on the assessment.

**Table 1:** Demographic and clinical characteristics of patients with Wilson’s disease

No	Sex	Age (years)	Education (years)	Length of illness (years)	Length of treatment (years)	History of psychiatric symptoms	Current neurological symptoms	Results of neuroimaging	Type of mutation
1	F	56	11	31	28	-	Tremor, speech disorder, ataxia	MRI: small lesions in frontal and parietal white matter; right GPi, general cortical atrophy	c.3207C > A p.H1069Q
2	M	70	17	40	34	-	Drooling, speech disorder, gait disorder, tremor	CT: general atrophy	3402delC p.A1135fs EX2 c.1847G > A R616Q
3	F	46	17	18	17	Behavioral disorders	Speech disorder, gait disorder, tremor	MRI: bilateral lesions in GP	c.3207 > A p.H1069Q
4	M	58	12	25	25	Depression	Speech disorder, gait disorder, tremor	MRI: atrophy of frontal lobes, atrophy of cerebellum (vermis, hemispheres)	c.3207 > A p.H1069Q
5	F	36	18	15	12	Anxiety disorder	Speech disorder	MRI: small lesions in white matter and corpus callosum, ventrices enlargement	c.3207C > A 4051C > T
6	M	28	13	8	4	Behavioral disorders, depression	Dysphagia, speech disorder, bradykinesia, tremor	MRI: bilateral lesions in GP, putamen, thalamus, pons, ventrices enlargement	c.3207 > A p.H1069Q
7	F	48	13	9	8	-	Speech disorders, spowolnienie, gait disorders, tremor	MRI: general atrophy, lesions in thalami and pons	c.3207 > A p.H1069Q
8	F	41	11	21	20	Unspecified mental disorders at age of 15	Drooling, speech disorder, gait disorder, tremor	MRI: general atrophy, bilateral lesions in GP and putamen	c.3207 > A p.H1069Q
9	F	46	13	26	20	-	Drooling, speech disorder, tremor	MRI: bilateral lesions in GPi, cerebral peduncles and red nuclei	c.3207 > A p.H1069Q
10	M	31	13	6	6	Cognitive and behavioral disorders	Speech disorder, involuntary movements	MRI: bilateral lesions in GP and putamen; frontal atrophy	c.3207 > A p.H1069Q
11	M	42	11	4	4	-	Speech disorder, involuntary movements, tremor, drooling, bradykinesia, gait disorders	MRI: bilateral lesions in GP, putamen, thalamus, pons	c.3207 > A p.H1069Q
12	F	47	11	7	7	Depression	Speech disorder, bradykinesia	MRI: cerebellar atrophy	c.3207 > A p.H1069Q
13	M	51	11	31	30	-	Speech disorder, drooling, dysphagia, gait disorders, tremor, involuntary movements	MRI: cerebral and cerebellar atrophy	c.2332C > G R778G EX2 -//-
14	F	44	12	9	9	Anxiety disorder	Drooling, tremor	MRI: bilateral lesions in GP, putamen, thalamus and pons	c.3207C > A p.H1069Q
15	M	60	15	26	26	-	drooling, speech disorder, tremor	MRI: bilateral lesions in GP, lesions in right SN and red nucleus; cerebral and cerebellar atrophy	c.3207C > A p.H1069Q
16	F	57	12	33	32	Cognitive disorder	Drooling, dysphagia, speech disorder, gait disorders, tremor	MRI: bilateral lesions in SN, red nuclei, GP and dentate nuclei	c.3207C > A 3818C > T
17	F	54	18	36	35	-	Tremor, speech disorder, drooling	MRI: cerebral and cerebellar atrophy	c.3207C > A p.H10969Q
18	F	44	13	6	5	-	Tremor, involuntary movements	MRI: bilateral lesions in GP, caudate nuclei, thalami and pons	c.3207C > A p.H1069Q
19	M	39	11	9	8	-	Tremor	MRI: bilateral lesions in putamina, thalami, pons and cerebellum	c.3207C > A p.H1069Q
20	M	39	11	11	9	-	Speech disorder, gait disorders	MRI: bilateral lesions in GP, thalamus, putamen; general atrophy	c.3207C > A p.H10969Q
21	M	44	15	20	17	-	Tremor	MRI: bilateral lesions in GPi, thalamus, cerebral peduncle and dentate nuclei	c.3207C > A p.H1069Q
22	F	50	13	8	7	-	Tremor, gait disorders	MRI: lesions in cerebellum, general atrophy	c.3207C > A p.H1069Q
23	M	23	13	4	3	Cognitive disorder	Drooling, dysphagia, speech disorder, gait disorders, tremor, dystonia, involuntary movements	MRI: bilateral lesions in putamen and pons	c.3207C > A p.H1069Q
24	F	51	12	14	12	-	Speech disorder, tremor, involuntary movements	MRI: cortical atrophy	c.3207C > A p.H1069Q EX2327t > C L776P
25	M	36	17	18	18	-	Dysphagia, speech disorder, rigidity	CT: lesions in basal ganglia; frontal atrophy	c.4051C > T p.Q1351X EX2 -//-
26	F	55	12	33	13	-	Tremor	MRI: bilateral lesions in GP, putamen, thalamus and pons; general atrophy	c.3207C > A p.H1069Q
27	F	47	12	29	24	-	Drooling, dysphagia, speech disorder, gait disorders, tremor, involuntary movements	MRI: lesions in putamina	c.3207C > A p.H1069Q EX 2 4051C > T p.Q1351X
28	F	25	14	11	4	-	Drooling, speech disorder, gait disorders, tremor	MRI: bilateral lesions in GP, putamen, caudate nucleus and thalamus	c.3207C > A p.H1069C EX2 3400delC
29	M	35	17	6	5	-	Speech disorder, tremor, involuntary movements	MRI: bilateral lesions in thalamus, pons, cerebellum; enlargement of ventricles	c.3207 C > A p.H1069C EX2 -//-
30	M	45	16	17	16	-	Speech disorder, tremor, involuntary movements	MRI: bilateral lesions in putamen, thalamus, cerebral peduncle, pons, dentate nuclei; atrophy of frontal cortex and cerebellum	c.3207 > A p.H1069Q

*CT* computed tomography; *GP* globus pallidus; *MRI* magnetic resonance imaging

Thirty age-matched healthy individuals were used as a control group. They were recruited from volunteers (persons visiting other patients hospitalized in the Department of Neurology). The inclusion criteria were (1) age 18–65 years and (2) no history of any neurological or psychiatric condition.

The characteristics of patients and controls are presented in Table 2. Magnetic resonance imaging (MRI) was available for 23 (77%) of the participants. In most cases, results showed increased signals primarily in the basal ganglia, specifically globus pallidus (*n* = 13; 57%), putamen (*n* = 7; 30%), and caudate (*n* = 4; 17%). In a few exams, changes were also observed in the thalamus (*n* = 3; 13%), cerebellum (*n* = 3, 13%), and/or pons (*n* = 3; 13%). In some patients, brain atrophy was reported, generalized (*n* = 5; 22%) or limited to the cerebellum (*n* = 6; 26%), as well as ventricular enlargement (*n* = 7; 30%). The patients exhibited various neurological symptoms ranging from mild to moderate, with the most common being dysarthria (*n* = 24; 80%) and tremor (*n* = 23; 77%). Less common were gait disturbance (*n* = 12; 40%) and/or drooling (*n* = 11; 37%). Next in order of frequency included dysphagia (*n* = 6; 20%), involuntary movements (*n* = 6; 20%), psychomotor slowing (*n* = 3; 10%), imbalance (*n* = 1; 3%), major change in mimic expression (*n* = 1; 3%), and rigidity (*n* = 1; 3%).

**Table 2:** Characteristics of WD patients and healthy controls

Group	Male/female ratio	Age (years) Mean (*SD*)	Education (years) Mean (*SD*)	Disease duration (years)^a^ Mean (*SD*)	Treatment duration (years)^b^ Mean (*SD*)
WD (*n* = 30)	16/14	44.9 (10.7)	13.5 (2.3)	17.7 (10.9)	15.3 (10)
Control (*n* = 30)	19/11	43.1 (13.2)	14.4 (2.8)	-	-

*SD* standard deviation; *WD* Wilson’s disease

^a^Duration of disease: the time between onset of first symptoms and diagnosis

^b^Treatment duration: the time between pharmacotherapy implementation and participation in the study

### Procedure

One experienced clinical neuropsychologist (M.R-G.) conducted the assessment of all study participants during individual sessions under optimal conditions. Informants were family members (most frequently, spouses) or close caregivers. The interview and questionnaire were conducted individually. An informed consent was obtained in all cases. The study was approved by the local Bioethics Committee. All medical information was derived from the neurological department’s WD database.

### Measurement tools

1. Addenbrooke’s Cognitive Examination-Revised (ACE-R) [22] is a screening tool of high diagnostic utility for the identification of cognitive impairment in different patient populations that has proven to be sensitive in early stages of cognitive regress [23].
2. Lille Apathy Rating Scale (LARS) has been proven to be useful, reliable, and valid in patients with basal ganglia disorders (e.g., PD) [18, 24]. LARS is a semi-structured interview designed in accordance with Marin’s definition of apathy [25]. It consists of 33 questions and responses are coded either on a three-point or five-point Likert-type scale. The items are classified into 9 domains: reduction of everyday productivity, lack of interest, lack of initiative, extinction of novelty seeking, lack of motivation, blunting of emotional responses, lack of concern, poor social life, and decreased level of self-awareness. There are also four subscales measuring main factors of apathy: intellectual curiosity, action initiation, emotion, and self-awareness. The global score of LARS ranges between − 36 and + 36, with higher values suggesting more severe apathy. Since the official caregiver version of LARS [26] was not available in Polish adaptation at the beginning of this study, the informants were asked rephrased questions contained in items 3–9 of the original LARS, referring to the patients’ subjective assessments. Items 1 and 2 were excluded due to the observational nature of these questions. Consequently, for more direct comparison, items 3–9 in both versions (LARS-self and LARS-proxy) were analyzed. These shortened scales showed reasonably high internal consistency (Cronbach’s α = 0.67 and 0.76, respectively), as measured in the healthy control group.
3. Dysexecutive Questionnaire (DEX), forming a part of the Behavioral Assessment of Dysexecutive Syndrome (BADS) [27], consists of 20 questions for subjective assessment (DEX-self). It focuses on the potential deficits in the emotional, motivational, behavioral, and cognitive domains that are characteristic for dysexecutive syndrome. There also exists a version for informants (DEX-proxy), which proved to be sensitive in the cases of patients with executive dysfunction [28]. Comparison of self-report and informant responses can be an initial indicator of patients’ self-awareness. In order to gain some insight into the nature of possible executive problems and to be able to investigate the relationships of different aspects of this cognitive domain and apathy, we further divided the general DEX scores into three components: inhibition, volition, and social regulation, according to the recent factor analysis by Shaw and collaborators [29].

### Data analysis

Data analyses were performed using R programming language. The differences between WD patients and healthy controls, in terms of both self-rated and proxy-rated LARS and DEX, were analyzed using two-way mixed ANOVA with group (WD or healthy controls) as a between factor and rater (self or proxy) as a within factor. Two healthy participants were excluded from the analysis of LARS results due to missing data (lack of LARS-proxy). Post hoc tests with Bonferroni correction were used to investigate specific differences if any effects were revealed in ANOVA. Any other differences between the two groups were tested using the independent t-test or Mann–Whitney U test. To investigate any other linear relationships of variables, either Pearson r or Spearman rho was calculated. In order to enable comparisons between the different analyses, r coefficient was consequently used as an index of effect size. The level of significance was set at *p* < 0.05.

## Results

### Comparison of demographic characteristics

Both groups were balanced in terms of age (*t*[58] = 0.602, *p* = 0.55, *r* = 0.08) and education (*U* = 367, *p* = 0.215, *r* =  − 0.16). There were more male participants in the healthy control group, but the difference was not significant (*chi*^*2*^[1] = 0.27, *p* = 0.6).

### Comparison of cognitive performance

WD patients performed significantly poorer than healthy controls on ACE-R (*Med* = 91.5, *IQR* = 8 and *Med* = 95.5, *IQR* = 4, respectively; *U* = 191, *p* = 0.001, *r* =  − 0.5). Since executive functioning might be of particular importance in this context, we also analyzed the total scores of the ACE-R’s verbal fluency task as a regard of good measure of executive domain [30]. The WD group obtained significantly lower scores than healthy controls (*Med* = 11, *IQR* = 2 and *Med* = 13, *IQR* = 1.5, respectively; *U* = 166, *p* = 0.001, *r* =  − 0.55) in this subtest.

### Comparison of LARS and DEX scores

**Fig. 1:**
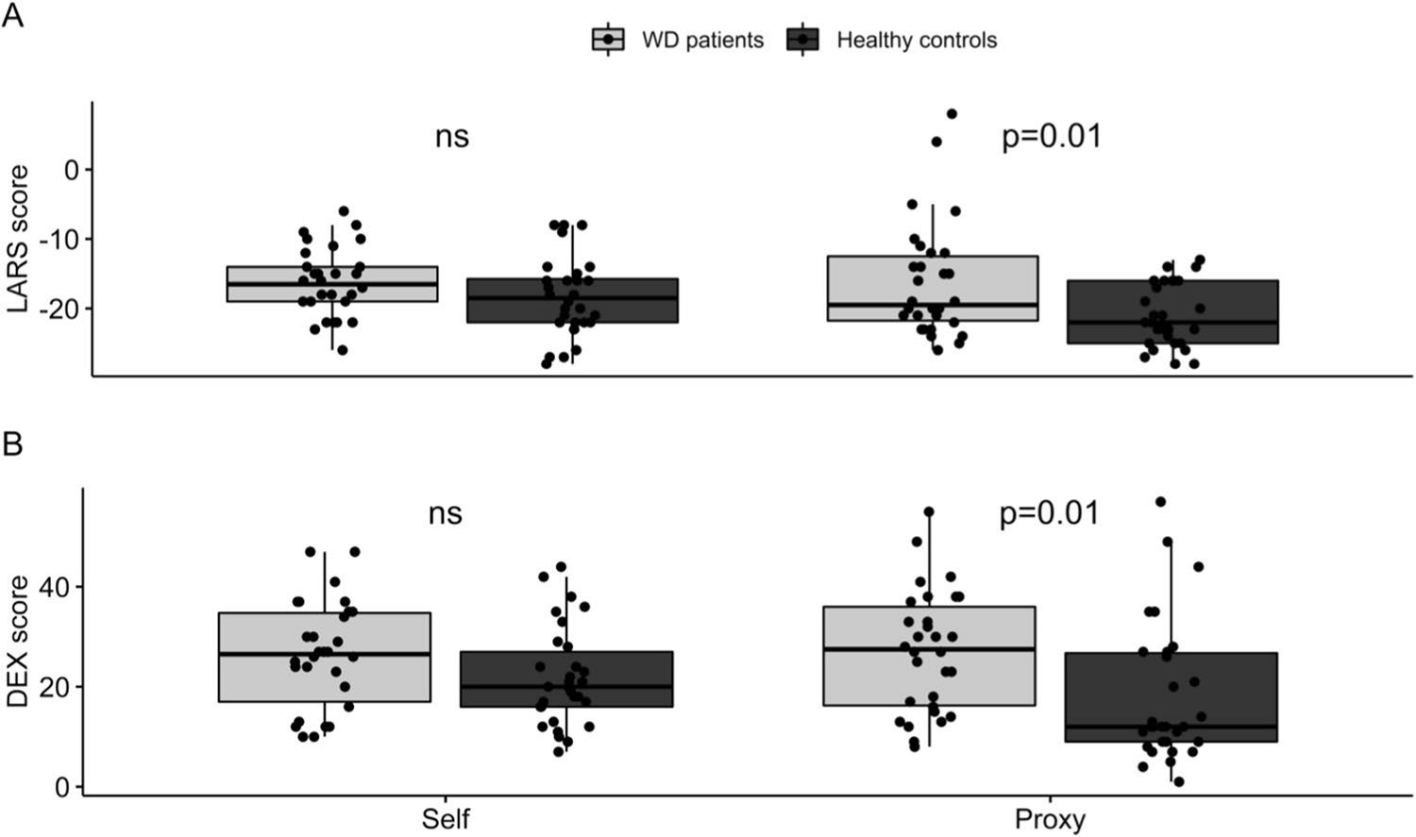
Comparison of LARS (**A**) and DEX (**B**) scores in a group of WD patients and healthy controls. Self: self-rating; Proxy: rating performed by a caregiver

ANOVA revealed significant main effects of group on LARS scores (*F*[1, 56] = 6.742, *p* = 0.012, *r* = 0.27). Post hoc tests clarified that this result could be attributed to the difference between proxy rates (*p* = 0.01) since the difference between self-ratings was not significant (*p* = 0.31; Fig. 1A).

Similarly, in the analysis of DEX, only group factor had significant impact on patients’ scores (*F*[1, 58] = 8.053, *p* = 0.006, *r* = 0.28). Again, the difference between proxy rates (*p* = 0.01) explained the abovementioned main effect while self-ratings were similar in both groups (*p* = 0.25; Fig. 1B).

### Relationships between cognition and apathy

In the WD group, LARS-self was related to neither ACE-R (*p* = 0.052) nor global DEX-self (*p* = 0.68). Similarly, there was no significant relationship of LARS-proxy in this group, either with ACE-R (*p* = 0.3) or with global DEX-proxy (*p* = 0.13). There was no significant relationship between LARS and DEX components in either version of these measures, except that between LARS-proxy and inhibitory component of DEX-proxy (*r* = 0.48, *p* = 0.007).

To further investigate the possible relationship between cognitive impairment and apathy symptoms, we divided the clinical group into a subgroup of nine (30%) patients fulfilling the ACE-R criteria for cognitive impairment (cut-off score 88) and those with higher scores. The Mann–Whitney U test did not reveal any significant differences between these two groups in any of the psychopathological measures except, not surprisingly, ACE-R verbal fluency (*U* = 169.5, *p* = 0.001, *r* = 0.64).

In an attempt to roughly evaluate a frequency of apathy in neurological WD patients, we selected patients who obtained scores higher than any of the participants from the healthy control group. In the case of LARS-self, there was only one patient (3%; no. 2) who met these criteria. However, when LARS-proxy was considered, the scores of eight patients (27%; nos. 2, 4, 6, 12, 20, 23, 25, 30) were abnormally elevated. Interestingly, among these patients was only one female. Four (50%) of these patients had previous history of psychopathology, including all three patients from the entire WD group who suffered from affective disorders in the past. Only three patients (37%) had ACE-R scores below the cut-off point. The range of illness duration in this subgroup was 4–40 years.

## Discussion

A clear tendency of patients with Wilson’s disease to exhibit symptoms of apathy is the most important finding of this study. Significantly, these symptoms are reported by patients’ caregivers and not by patients themselves. The same pattern applies to self-rated symptoms of executive dysfunction. It is very likely that due to poor insight, patients underestimated their psychopathological symptoms. It was estimated that roughly 30% of patients were likely to suffer from some degree of apathy since their scores in LARS-proxy were higher than that of any of healthy controls.

There are numerous reasons for emergence of apathy in WD. The main anatomical correlates of apathy are pathological changes in the ventral striatum, dorsal anterior cingulate cortex, and other brain regions connected to the abovementioned [13]. Bhatia and Marsden [31] reviewed 240 cases of patients with lesions in the basal ganglia and concluded that 13% had relatively isolated significant adynamia, and of the latter, 70% were patients with caudate pathology. Severe apathetic-abulic disorders were also described in bilateral ischemic strokes involving the medial parts of the thalamus [32] and, as a consequence of damage to the medial parts of the frontal cortex [33], apathy was typically considered. All of these brain structures are susceptible to damage in the course of WD.

Changes in neurotransmission may be another important cause of apathy [34]. In patients with WD, dopamine deficiency—most likely associated with copper deposition in the basal ganglia—may play a crucial role in inducing apathy [35]. Although the reduction in dopaminergic projections is not as prominent as in PD, a recent study by McGuigan et al. [36] revealed that modulation of dopamine levels may reduce some symptoms of apathy.

As suggested by previous studies, the key difficulty in measuring apathy symptoms in WD is poor insight of patients into their mental functioning [37, 38]. Therefore, the use of the so-called self-report measurement tools does not seem to produce fully reliable results. Patients’ limited self-awareness may lower the credibility of the interview and sometimes interfere with understanding and adherence to medical recommendations, which is essential for effective treatment. Self-description methods require the examined patient to have relatively intact metacognitive functions, i.e., insight into their own functioning, self-awareness, and ability to recognize their own deficits and preserved functions.

Self-awareness deficits may vary in intensity, from anosognosia to underestimating difficulties, and affect many or only selected domains of functioning. In the study by Seniów et al. [38], patients with the neurological form of WD rated their aggressiveness and interpersonal hypersensitivity in the Hopkins Symptoms Check List as lower than healthy controls, although increased irritability and conflictuality were usually emphasized (in the opinions of the patients’ relatives). Furthermore, patients with the neurological form of WD seemed to respond less emotionally to their severe, chronic illness with motor disability compared to other chronic diseases, e.g., rheumatoid arthritis [39].

Therefore, more objective methods are needed to effectively detect and assess symptoms of apathy. We believe that evaluations performed by a family member/caregiver are more reliable (although not fully objective) than simple self-reports and can provide important information in this aspect of a patient’s functioning.

Based on caregiver reports (LARS-proxy), it was found that apathy signs were independent of cognitive functioning. Individuals with clear cognitive impairment as measured by ACE-R (a slightly higher percentage of cognitive impairment than reported by Carta et al. [40] and Frota et al. [41] in the WD population) did not differ from the rest of patients in terms of the scores on descriptive clinical scales. This might be quite surprising since apathy is sometimes regarded as one of the aspects of executive dysfunction [42]. The relationship of WD and associated basal ganglia pathology with executive dysfunction is rather unquestionable in light of recent research [43]; however, research on the neurological form of WD shows that movement, cognitive, and emotional/motivational disorders are not strongly related. Although they often coexist, the degree of the deficit in each of the areas mentioned does not correlate with the severity of dysfunction in another [44]. In addition, psychopathological symptoms sometimes persist despite pharmacotherapy that is effective for the somatic (including motor) aspects of the disease.

It would appear that cognitive, emotional, and motivational changes in patients with WD could be partially related to subtle structural and functional abnormalities. These are often revealed by more sensitive and specific imaging techniques such as single-photon emission computed tomography [45, 46] or resting-state functional MRI [47]. Such techniques typically reveal pathological changes within a widespread network of both gray and white matter structures, which disrupt the functional connectivity crucial for cognitive, emotional, and motivational control [48].

Therefore, apathy seems to be another relatively independent area of psychopathology in WD. It is important to note, however, that until we have access to objective and sensitive measurement tools for these symptoms, all conclusions from studies using self-report and caregiver-report methods need to be treated with great caution.

## Data Availability

Not applicable.
